# Pathogenesis and Mechanism of Gastrointestinal Infection With COVID-19

**DOI:** 10.3389/fimmu.2021.674074

**Published:** 2021-11-10

**Authors:** Hao Zhang, Bo Shao, Qin Dang, Zhuang Chen, Quanbo Zhou, Hong Luo, Weitang Yuan, Zhenqiang Sun

**Affiliations:** ^1^ Department of Colorectal Surgery, The First Affiliated Hospital of Zhengzhou University, Zhengzhou, China; ^2^ Academy of Medical Sciences, Zhengzhou University, Zhengzhou, China; ^3^ Department of Hepatobiliary and Pancreatic Surgery, Guangshan County People’s Hospital, Xinyang, China

**Keywords:** angiotensin-converting enzyme 2 (ACE2), gastrointestinal tract, gut–lung axis, cytokine storm, coronavirus disease 2019 (COVID-19), RAS system

## Abstract

As a new infectious disease, COVID-19 is spread through the respiratory tract in most cases. Its source and pathological mechanism are not clear. The most common clinical feature is pulmonary infection. Also, a lot patients have gastrointestinal symptoms. Angiotensin-converting enzyme 2 (ACE2) is a functional cellular receptor for SARS-CoV-2, which is like SARS-CoV, a coronavirus associated with severe acute respiratory syndrome (SARS) outbreak in 2003. The tissues and cells expressing ACE2 are potential targets for SARS-CoV-2 infection, and the high expression of ACE2 in intestinal epithelial cells marks that SARS-CoV-2 may directly infect intestinal epithelial cells. Recent studies also suggest that SARS-CoV-2 existed and replicated in intestinal environment for a long time. The interaction between SARS-CoV-2 and RAS system leads to the decrease of local anti-inflammatory ability. The virus cycle leads to excessive imbalance of immune response and cytokine release. The downregulation of ACE2 after viral infection leads to gastrointestinal dysfunction. The above are the causes of gastrointestinal symptoms. Here, we reviewed the possible causes and mechanisms of gastrointestinal symptoms caused by COVID-19. Additionally, we discussed the influence of gastrointestinal symptoms on the prognosis of patients.

## Introduction

Since the end of 2019, COVID-19 has begun to spread rapidly around the world (1). Recent studies showed the persistent positive rate of SARS-CoV-2 in stool specimens of patients with COVID-19 (2). After a negative respiratory test, the continuous detection of SARS-CoV-2 in stool specimens or anal swabs takes a long time. The duration of virus positive in stool specimens may be longer than that in respiratory specimens (3, 4). Not only that, the results of Lin et al.’s research also showed that some patients (27.3%) did not have any lung imaging features of COVID-19, but had gastrointestinal symptoms, indicating that gastrointestinal tissues are susceptible to SARS-CoV-2 (5). It is very necessary for gastroenterologists to understand and explore the prevalence, pathogenesis, and clinical characteristics of SARS-CoV-2 infection in the gastrointestinal tract. Based on the current research between SARS-CoV-2 and the gastrointestinal mucosa, this article speculates about how SARS-CoV-2 causes gastrointestinal symptoms. Finally, we briefly summarize the gastrointestinal symptoms of COVID-19 patients and their impact on the prognosis of the disease.

## How SARS-CoV-2 Infects Human Tissue Cells

Current research shows that angiotensin-converting enzyme 2 (ACE2) is the receptor for SARS-CoV-2 (6, 7). ACE2 is expressed in human lung and intestinal epithelial cells (7, 8), and its expression in the intestine is more than four times that of other tissues (9). Also, ACE2 is highly expressed in proximal and distal small intestine cells (10). Through the detection of stool samples of confirmed cases, it was found that the detection rate of SARS-CoV-2 nucleic acid in stool samples was the same as the detection rate of throat swab samples (11).In the study of Xiao et al., infectious SARS-CoV-2 was isolated from stool, and positive staining of ACE2 and SARS-CoV-2 was observed in patients with positive stool test (12). Research shows that SARS-CoV-2 viruses can survive at a wide range of pH values at room temperature. The highly viscous mucus in the gastrointestinal tract protects viral RNA and virus particles, allowing the virus to retain its infectivity. Then, the virus is excreted with the feces (13, 14). Inflammatory conditions in the gastrointestinal tract disrupt the multi-layer barrier system and increase the expression of ACE2 in the intestinal epithelium. This allows SARS-CoV-2 to enter the intestinal epithelial cells (15). These can provide support for the hypothesis of fecal–oral transmission of SARS-CoV-2. Other than the fecal–oral route, an alternative route of viral entry to the GI cells may be through the tissue microvasculature. This will be further elaborated below.

However, whether SARS-CoV-2 can be transmitted through the fecal–oral route is still controversial. Infectious virus was isolated from intestinal tissue but not fecal specimens (16). Jeong et al. failed to directly prove the presence of viable SARS-CoV-2 in stool samples by cell culture isolation (17). Detection of high copy numbers of viral RNA in the stool does not equate to shedding of infectious viruses or transmission of the disease (18). Respiratory transmission was not specifically blocked, making it difficult to attribute the transmission to the fecal–oral route. Is the fecal viral load sufficiently high for human transmission? How long can the excreted virus persist in the environment? Can fecally shed virus infect animals that may serve as a reservoir for spread? During transmission, can gut be the first site of infection or does the virus spread to the gut from the respiratory or other tissues (18)? These all require more specific experimental demonstrations.

## The Mechanism of SARS-CoV-2 Causing Gastrointestinal Symptoms

### The Characteristics of SARS-CoV-2 Infection of Intestinal Cells and the Interaction With ACE2

SARS-CoV-2 infection of intestinal cells mainly has the following two characteristics. First, the immune response induced by SARS-CoV-2 is different. Under *in vitro* conditions, SARS-CoV-2 was observed to infect human intestinal epithelial cells and mesenteric cells, leading to their apoptosis (19). The replication efficiency of SARS-CoV-2 is lower than that of SARS-CoV. The cytopathology induced in human intestinal epithelium is relatively less, and it induces a stronger innate immune response, including more powerful interferon and pro-inflammatory response (20). Second, lung infections can be accompanied by gastrointestinal infections, which can lead to recurrence of the disease. For patients who experienced recurrence, the phylogenetic analysis of the full-length SARS-CoV-2 genome in the gastrointestinal tract showed that the virus detected in the positive retest evolved from the original parent virus (21). Some patients still have a positive stool test when the throat swab test is concealed (22–24).This suggests that SARS-CoV-2 exists and replicates at a low level in the intestine for a long time (21). This is because in the early stage of new coronavirus infection, it is difficult for a small number of patients to quickly obtain immunity to SARS-CoV-2 and produce targeted IgM or IgG, which eventually causes the virus to persist in the gastrointestinal tract for a long time (25, 26). A recent study pointed out that the virus can hide in the mesenteric tissue and infect the lungs through the vasculature, causing disease (27).

**Figure 1:**
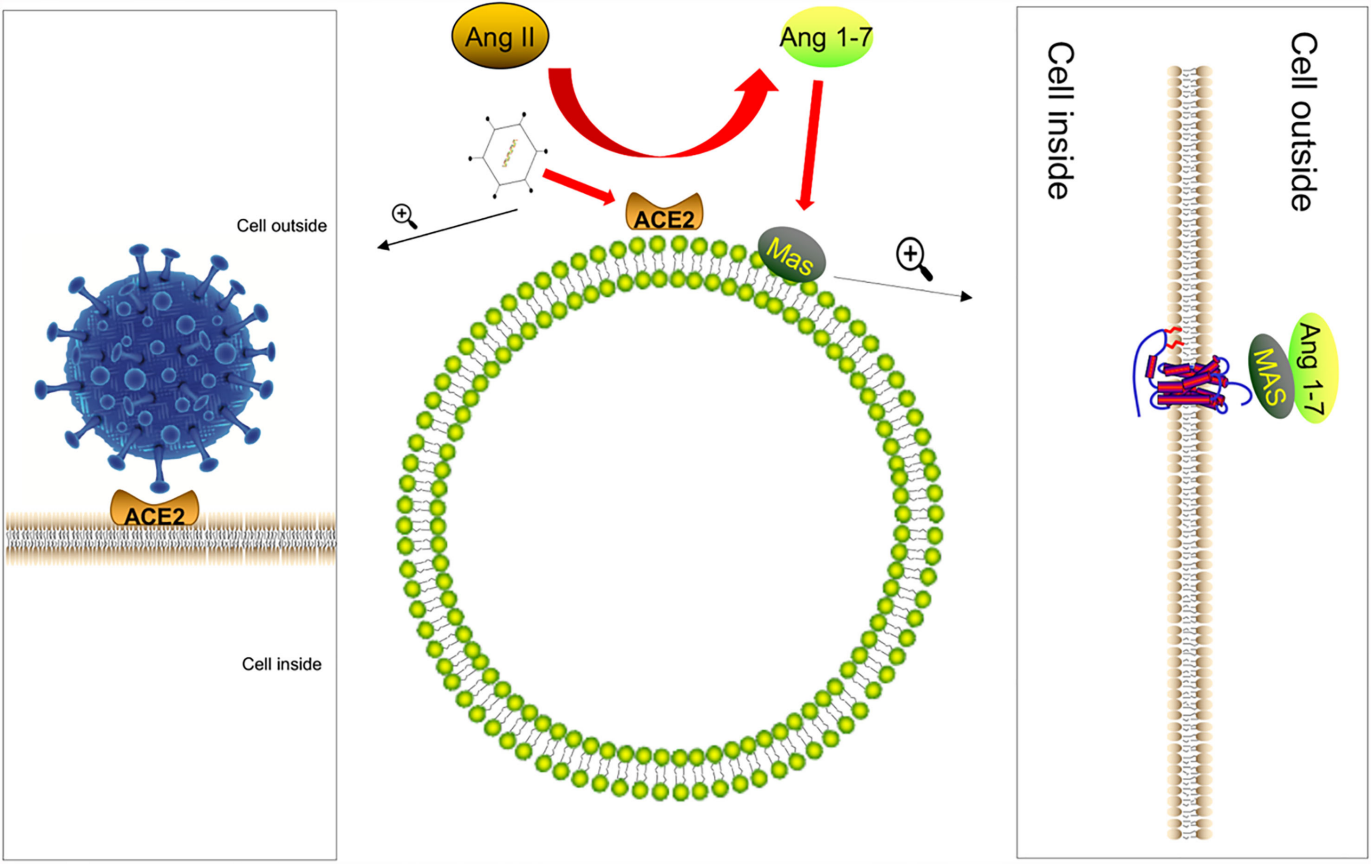
The S protein of COVID-19 infects the host by combining with ACE2. After the virus infects the host, the expression of ACE2 protein is downregulated. ACE2 converts Ang II, the main biologically active molecule of RAS, to Ang 1-7; Ang (1Mel7) has anti-inflammatory and anti-remodeling effects after binding to the Mas receptor and coupling with Gq protein. Therefore, after the virus infects the host, it is likely to cause the intestinal anti-inflammatory ability to decrease.

We know that the renal angiotensin system (RAS) regulates systemic or local body functions and plays an important role in regulating blood pressure, electrolytes, organ functions, etc. (28). RAS is regulated by the opposing actions of two key carboxypeptidases, angiotensin-converting enzyme (ACE) and ACE2 (29, 30). Angiotensin II (AngII) is a vasoactive substance that can raise blood pressure. AngII is a vasoconstrictor, its overproduction causes inflammation (31, 32), and ACE2 is an enzyme that converts AngII, the main biologically active molecule of RAS, into Ang 1-7 (33, 34). The physiological significance is to exert anti-inflammatory and anti-remodeling effects (35). Recent reports showed that the S protein of COVID-19 would use the same receptor ACE2 as SARS-CoV to infect the host (36–39). Furthermore, the expression of ACE2 protein was downregulated after the virus infects the host (7). The lack of ACE2 was due to the internalization of the SARS-CoV-2–ACE2 complex, which limited the role of ACE2 as the carboxypeptidase of AngII and desarginine-bradykinin and other polypeptide hormones (40, 41). Based on this, we speculate that COVID-19 will cause the downregulation of ACE2 in the intestinal mucosal cells during the infection process, leading to the disorder of the RAS system and the decline of the anti-inflammatory ability of the intestinal mucosa (**Figure 1**). This is one of the reasons why people infected with the new coronavirus have intestinal symptoms.

There are very few reports about SARS-CoV-2 infection leading to viremia (42, 43). Recently, in a case, researchers isolated and cultured a live SARS-CoV-2 virus strain from the stool of an advanced patient and believed that SARS-CoV-2 might be able to migrate from the lungs to the blood circulation to the digestive tract (44). We agree with this view, but when viremia is present, patients often have severe systemic inflammation and cytokine storms (45). We believe viremia may be a stage that causes intestinal symptoms in patients, but it is not the main cause of intestinal damage.

### Immediate Immune Response and Inflammatory Factor Storm Caused by SARS-CoV-2 Infection and Intestinal Tissue Damage

In the early stage of SARS-CoV-2 infection, the body’s immediate immune response to the virus can effectively clear up the virus (46). Stimulation of innate immune cells leads to secretion of inflammatory mediators, which together with complement system exert antiviral effects in the early stage (47). Viruses evolved to have various strategies to circumvent the innate immune response. For example, viruses can evade the complement system by removing antibody–antigen complexes from cell surfaces, decreasing Fc receptor expression, or by mimicking the complement regulatory component (48, 49). Studies have shown that the replication efficiency of SARS-CoV-2 is lower, and the natural immune response (including the activation of type I and type III interferons) induced in human intestinal tissue is stronger than SARS-CoV (20). Due to the complex virus–innate immune interaction, the immune system may sometimes delay recovery, eventually leading to the patient’s disease progression and even death (46).

In colonic epithelial cells expressing ACE2 positively, viral infection was significantly enhanced (50). After endocytosis, the positive-sense RNA highjacked cellular machinery for viral RNA and viral-specific protein synthesis. Viral particles were then assembled in the cellular cytoplasm and released into the gastrointestinal (GI) tract (51). In the lungs, due to the presence of ACE2 receptors, blood vessels in the lungs can easily become targets, triggering microthrombosis, immune complex deposition, and excessive humoral immune responses, leading to subsequent hypoxic damage to various important organs (52). From this, we speculate that the virus spreads through fecal–oral transmission to reach the gastrointestinal tract. In the intestine, virus particles reach ACE2-positive intestinal epithelial cells under the protection of the mucus layer (15). Infection of intestinal epithelial cells leads to the release of virus particles. The virus particles then reach the rich vascular network in the submucosa, eventually leading to local inflammation of the intestinal tract and secondary intestinal damage. In *in vitro* experiments, SARS-CoV-2 can infect and replicate effectively in isolated human intestinal tissues by releasing infectious virus particles, and the innate immune response induced in human intestinal tissues is stronger than SARS-CoV (53). Here, we will discuss the inflammatory response mechanism caused by SARS-CoV-2 infection that may lead to intestinal cell damage.

Increased levels of cytokines often occur in critically ill patients with COVID-19, including IL-6, IL-2R, IL-10, and tumor necrosis factor-α (54). An autopsy report showed that in patients with fatal new coronary pneumonia, there was a widespread systemic inflammation involving the gastrointestinal tract, and neutrophils and reticular structures persist. However, SARS-CoV-2 infected cells were only occasionally seen in the late stage of new coronary pneumonia. This suggested a maladaptive immune response (55). In this maladaptive immune response, the direct cytopathological effects of SARS-CoV-2 induced apoptosis (a highly inflammatory form of programmed cell death) and release endogenous danger signals (56), which were local and remote recognition by epithelial cells, macrophages, and endothelial cells. A cascade of local inflammation ensued, characterized by secretion of pro-inflammatory cytokines and chemokines that attracted monocytes, macrophages, and T cells that mediated extensive pathology, culminating in tissue damage (57). When local inflammation occurs, macrophages frequently communicate with SARS-CoV-2 targets through chemokines and phagocytic signals (58), and inflammation may upregulate ACE2 expression on macrophages (59), further amplifying viral sensing and tissue injury (57). Postmortem analyses of secondary lymph nodes and spleen implicated CD169+ macrophages that contain viral nucleocapsid protein as mediators of activation-induced cell death (AICD) of lymphocytes (60). A recent study pointed out that SARS-CoV-2 induces IL-1 production in macrophages and mast cells (MCs), thereby inducing gene expression and activating other pro-inflammatory cytokines. Since IL-1 is toxic, IL-1 produced by ubiquitous MCs and macrophages activated by SARS-CoV-2 also causes gastrointestinal diseases. In addition, IL-1 also promotes the release of nitric oxide and the release of inflammatory arachidonic acid products such as prostaglandin and thromboxane A2 (61). All these effects will promote the generation and development of cytokine storms and lead to secondary intestinal damage.

In addition, patients with severe new-onset coronary pneumonia have significantly higher levels of IL-6 than patients with mild pneumonia (50). This may be because certain viral products (such as human immunodeficiency virus TAT protein transactivator) enhanced the DNA binding activity of nuclear factor κB (NF-κB) and nuclear factor IL-6 (NF-IL-6), thereby increasing IL-6 mRNA transcription (62, 63). IL-6 is usually synthesized locally in the acute phase of inflammation and mediates the multidirectional effects of immune response and hematopoiesis (63). This cytokine also promotes the specific differentiation of normal CD4+ T cells, which is necessary for T-helper 17 (Th17) to differentiate from simple CD4+ T cells, and is related to immune tolerance, autoimmunity and chronic inflammatory diseases (64). The other reason is a potential dysregulation of the AngII–AT1R pathway downstream of ACE2. AngII not only acted as a vasoconstrictor, but also acted as a pro-inflammatory cytokine through AT1R (65). SARS-CoV-2 activated NF-κB and STAT3 through the AngI–AT1R pathway after SARS-CoV-2 infection, and then activated IL-6 amplifier (IL-6 Amp), which was a mechanism for STAT3 to overactivate NF-κB and led to various inflammations and autoimmune diseases (66). A meta-analysis showed that the inflammatory cytokines including IL-6, IL-10, and TNF-α were intensively increased in patients with diarrhea (67). Therefore, there is excessive activation of IL-1, IL-6, and NF-κB in the intestinal system, leading to various damages to the gastrointestinal tract.

### ACE2-Mediated Gastrointestinal Microbiota Imbalance and Intestinal Epithelial Injury

Previous studies showed that the incidence of gastrointestinal symptoms in patients with COVID-19 was approximately 61%. Symptoms such as diarrhea, vomiting, and abdominal pain were reported (5). If the presence of SARS-CoV-2 is detected in the gastrointestinal tract tissue, this usually means that the symptoms are severe. Another study also showed that the incidence of abdominal pain in patients entering the intensive care unit was significantly higher than that in patients who did not need to enter the intensive care unit (68). SARS-CoV in 2003 used the human ACE2 receptor to infect the host, and a considerable number of patients developed gastrointestinal symptoms (69). Recent studies reported that SARS-CoV-2 used ACE2 receptors more effectively than SARS-CoV (70). Therefore, we cannot ignore the effect of SARS-CoV-2 on ACE2 in the gastrointestinal tract.

ACE2 regulates the expression of the intestinal neutral amino acid transporter, which is very important for the composition of intestinal microbiota (71). Therefore, diarrhea and other gastrointestinal symptoms in patients with COVID-19 are likely to be related to the downregulation of ACE2 expression. Here, we summarize the possible causes and mechanisms of the effects of COVID-19 on the gastrointestinal tract. Studies showed that the transepithelial absorption of amino acids through intestinal epithelial cells referred to the sequential transport process through the lumen, brush border, and basolateral membrane, and the step of amino acids passing through the lumen required the mediation of various amino acid transporters.

**Figure 2:**
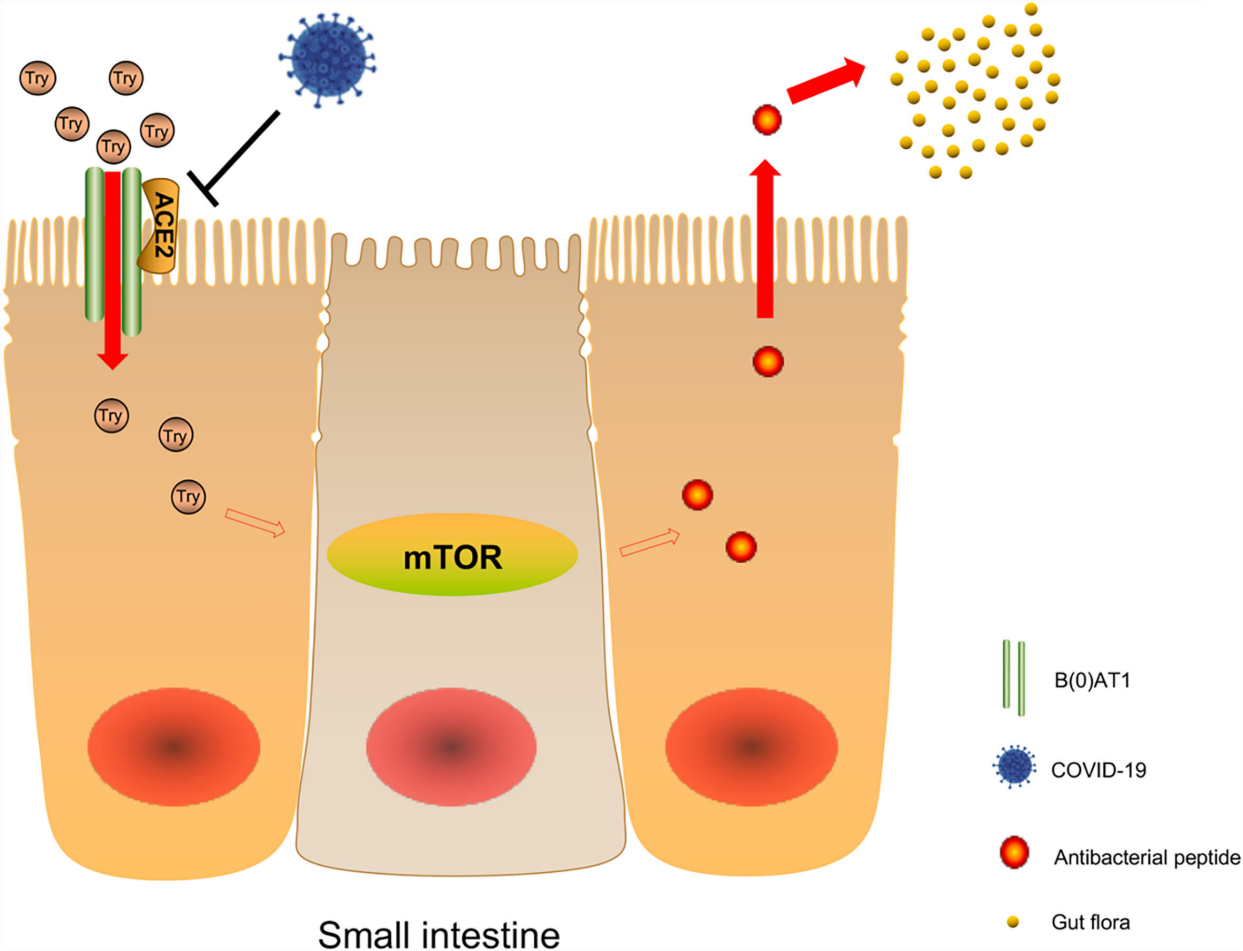
Possible mechanism of COVID-19 affecting intestinal microbiota. Sodium-dependent neutral amino acid transporter B(0)AT1 is expressed in small intestinal cells and plays an important role in amino acid absorption, while the expression and function of B(0)AT1 depend on the existence of ACE2. After viral infection, the decrease of ACE2 expression will lead to the decrease of B(0)AT1 expression, and dietary tryptophan is mainly absorbed through the B0AT1/ACE2 transport pathway on the surface of the small intestine epithelium, resulting in a significant decrease in plasma tryptophan levels, and lack of tryptophan and its metabolite nicotinamide leads to a decrease in mTOR pathway activity, which affects the expression of antibacterial peptides in the intestinal flora.

Studies reported that sodium-dependent neutral amino acid transporter B(0)AT1 was expressed in small intestinal cells and plays an important role in amino acid absorption, while the expression and function of B(0)AT1 depend on the existence of ACE2 (72). Hartnup disease is a rare autosomal recessive disease caused by B(0)AT1 gene mutation (73, 74). The disease causes obvious symptoms of diarrhea. Tatsuo et al. found that there was no change in the morphology and ultrastructure of the small intestine and large intestine in ACE2 knockout mice. However, after stimulation with sodium dextran sulfate (SDS), the intestines of ACE2 knockout mice showed more obvious inflammation, weight loss, and severe diarrhea (71). B(0)AT1 cannot be expressed in the intestines of ACE2 knockout mice, but dietary tryptophan is mainly absorbed through the B(0)AT1/ACE2 transport pathway on the surface of the intestinal epithelium. As a result, the level of plasma tryptophan decreased significantly, while the deficiency of tryptophan and its metabolite nicotinamide decreased the activity of the mTOR pathway (71, 75). The mTOR pathway regulates the expression of antimicrobial peptides that affect intestinal microbiota. Changes in antimicrobial peptides will affect the ecology of large and small intestinal microbiota and cause local enteritis and diarrhea (71). Combined with a recent autopsy report of COVID-19 patients, intestinal injury was not obvious to the naked eye, and the small intestine showed phased dilatation and stenosis. Intestinal injury was probably caused by intestinal inflammation. The Susanna Nikolaus team studied 500 patients with inflammatory bowel disease and found that tryptophan deficiency promotes the development of inflammatory bowel disease and exacerbates disease activity (76). Therefore, we infer that the gastrointestinal symptoms in some patients with COVID-19 may be due to the downregulation of ACE2 expression, which affects the disturbance of tryptophan absorption and local enteritis caused by the ecology of intestinal microbiota through the above pathway (**Figure 2**). B(0)AT1 was also shown to interact with another coronavirus receptor, aminopeptidase N (APN) (77). All these findings suggest that B(0)AT1 may have a certain regulatory effect on the ability of some coronaviruses to infect the intestine.

A recent study pointed out that several biosynthetic pathways, including the tryptophan biosynthetic pathway, will change in COVID-19 patients. This may be mainly caused by intestinal microbes. The study also found the depletion of several low-water-soluble long-chain fatty acids or fatty alcohols, such as arachidic acid, behenic acid, and 1-hexadecanol, in the feces of COVID-19 patients (78). The abovementioned microorganism-derived metabolites are closely related to regulating the host’s inflammatory response and promoting tolerance and drug resistance to viral pathogens (79, 80). Understanding how SARS-CoV-2 causes changes in the intestinal microbiota and fecal metabolites is of great significance to the study of the mechanism of SARS-CoV-2 causing gastrointestinal symptoms.

### Thrombosis and Coagulopathy, Sepsis, and Gastrointestinal Injury

Some recent cases reported repeated diarrhea, gastrointestinal bleeding, and acute mesenteric thrombosis in patients infected with the new coronavirus (81, 82). According to a recent study, bowel abnormalities were a common finding (31%) during abdominal imaging of patients with COVID-19, and patients who required laparotomy often had histological ischemia due to small blood vessel thrombosis (83). An imaging study showed that in patients without COVID-19, the left flexure colon and sigmoid colon were at the highest risk of ischemic colitis, while the distal rectal segment usually survived due to its double blood supply, and in patients with COVID-19, seven cases have reported thickening of the colon/rectum. This difference in the location of the disease also reveals the relationship between intestinal ischemic injury and SARS-CoV-2 infection (83, 84). A case report also confirmed that the gastrointestinal bleeding of patients with COVID-19 was caused by SARS-CoV-2 infection (85), and it was considered to be a thrombotic dysfunction caused by excessive inflammation, hypoperfusion, and even direct inflammation (86). Recent studies also reported patients who died of thrombotic diseases, or were infected with the new coronavirus with venous thromboembolism (VTE) and peripheral arterial thrombosis (60, 87, 88). Here, we summarize the relevant mechanisms of intestinal coagulation diseases caused by SARS-CoV-2.

ACE2 and TMPRSS2 (a serine protease) are expressed in endothelial cells (89, 90), and the S protein of the virus can also induce the downregulation of ACE2 (7). The downregulation of ACE2 is related to the local increase of Ang II (7). The increase of Ang- has a pro-inflammatory effect, leading to increased blood tumor necrosis factor-α and interleukin-6 levels (91). Both cytokines promote the rupture of the endothelial barrier (92) and reduce the level of Ang-(1-7) (93), leading to the occurrence of sepsis (94). Sepsis increases the level of inflammatory factors in the blood. Many cytokines are related to the production of blood’s procoagulant state. For example, the increase of IL-6 level is related to the increase of fibrinogen level (95). Increased levels of cytokines also led to tissue damage, thrombotic microangiopathy, endotheliitis, and endothelial dysfunction (96). The direct attack of SARS-CoV-2 on the vascular endothelium eventually led to a vicious cycle between sepsis and increased cytokine levels. The damage of vascular endothelial cells and the increase of cytokines in the blood together led to the hypercoagulable state of the blood, which induced the occurrence of intestinal coagulation diseases.

**Figure 3:**
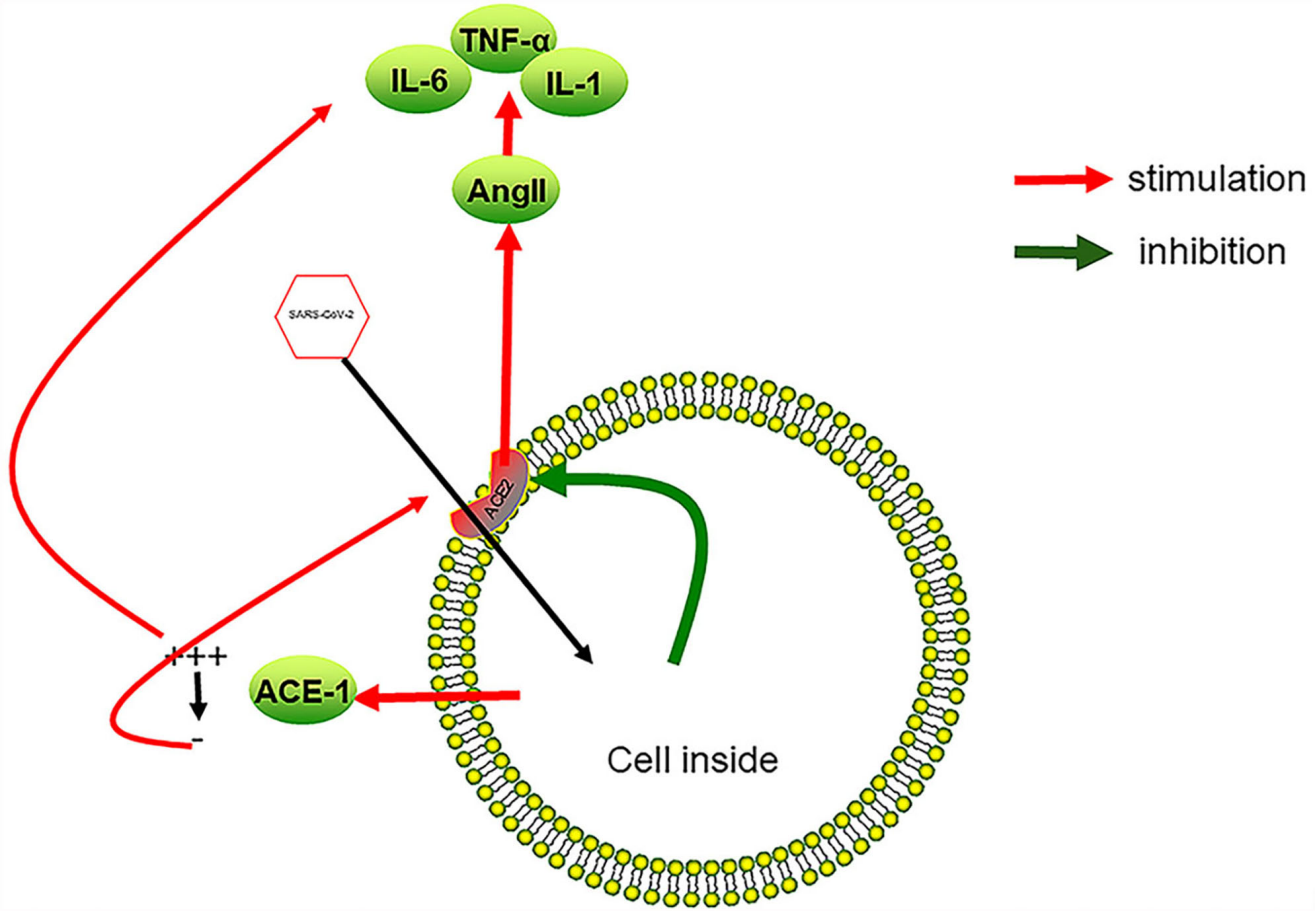
SARS-CoV-2 infection causes inflammation of vascular endothelial cells. After SARS-CoV-2 enters endothelial cells from ACE2, it promotes the reduction of ACE2 on the cell surface, which in turn leads to an increase in AngII in the blood, a decrease in local anti-inflammatory ability, and an increase in the expression of cytokines such as TNF-α, IL-6, and IL-1. Overexpression of inflammatory factors leads to the damage of vascular endothelial cells and the formation of a local procoagulant state. On the other hand, SARS-CoV-2 causes ACE1 to fall off the cell surface at the initial stage of infection, which promotes local plasma leakage and the expression of inflammatory factors. As the free ACE1 drops to a very low level, ACE2 is induced, which, in turn, promotes SARS-CoV-2 to enter endothelial cells. A vicious cycle between SARS-CoV-2 infection and overexpression of inflammatory factors forms.

In addition, studies showed that SARS-CoV-2 connected through the ACE2 receptor and enters cells, causing local inflammation and activating endothelial cells (97). The activation of lung endothelial cells led to the shedding of ACE1, which was described as the massive release of ACE1 from the cell membrane, followed by a rapid increase in the level of AngII, leading to inflammation, coagulation and capillary leakage (98). Here, we make an assumption: ACE1 shedding may also occur in infected intestinal endothelial cells. When the free ACE1 in the blood disappears, AngII will also drop to a very low level. Decreased levels of AngII can induce ACE2 synthesis, and may cause more SARS-CoV-2 to enter tissue cells (99) (**Figure 3**).

The increased levels of D-dimer and fibrinogen in many patients with COVID-19 may not only be a common cause of peripheral and pulmonary thrombosis, but also be the main cause of intestinal hypercoagulability and ischemic events (100, 101). Among patients hospitalized with COVID-19, the proportion of patients with D-Dimer increased as high as 47% (102). Compared with patients with coagulopathy, the most typical manifestation of patients with COVID-19 was increased D-dimer concentration (101). IL-6 induced mononuclear cells to express tissue factor, which in turn caused coagulation activation and thrombin generation. TNF-α and IL-1 are the main mediators that inhibit the endogenous anticoagulation pathway (103). Therefore, it is necessary to detect the levels of D-dimer and IL-6, TNF-α, and IL-1 to prevent and predict the intestinal coagulation disease of COVID-19. Polyphosphates are derived from microorganisms; activate platelets, mast cells, and factor 12 (FXII) in the coagulation pathway; and play other downstream roles in enhancing the procoagulant reaction of the endogenous coagulation pathway (104). The imbalance of gastrointestinal microbiota and the influence of gastrointestinal microbiota metabolites on the complications of intestinal thrombosis is also a point worth studying.

## The Gastrointestinal Symptoms of COVID-19 Patients and Its Influence on the Prognosis of the Disease

A number of large-scale case studies reported the clinical characteristics of patients with COVID-19 infection. In addition to the most common symptoms of fever and cough, each case study report also had gastrointestinal symptoms (105–112). Due to differences in institutions and regions, and the lack of a unified standard for judging gastrointestinal symptoms, the incidence of gastrointestinal symptoms in these studies is not the same. The most common of these was the case study of Zhang’s team; 39.6% of patients had gastrointestinal symptoms. In studies that include more cases of severely ill patients, the incidence of gastrointestinal symptoms has increased significantly. The symptoms included nausea, diarrhea, loss of appetite, abdominal pain, hiccups, and vomiting (111). Some cases were even accompanied by gastrointestinal bleeding (113, 114), intussusception (5, 115, 116), emphysema (117), and acute intestinal ischemia (118, 119). In some cases, severe gastrointestinal symptoms were the first manifestation, such as rectal perforation (120), acute pancreatitis (121), gastrointestinal bleeding (122), and colitis (123). This may be related to the underlying disease of the gastrointestinal tract (124). Gastrointestinal symptoms can be the first manifestation of COVID-19 infection. The underlying mechanism may be due to the expression of ACE2 in intestinal epithelial cells, making the digestive system a potential route of infection (125). Gastrointestinal symptoms have a certain familial clustering. A study from Bangladesh showed that if patients had gastrointestinal symptoms, their family members were at increased risk of infection and are more likely to develop gastrointestinal symptoms (126).

Diarrhea often indicated a severe type of inflammation, so it was more important for the prognosis of patients (67, 116, 127). Xi’s case–control study pointed out that patients with gastrointestinal symptoms had a significantly higher chance of developing critical cases than patients without gastrointestinal symptoms (128), and they were more likely to develop critical diseases such as ARDS, liver injury, and shock (129). In critically ill patients, the inflammation caused by viral infection was severe, the intestinal damage was aggravated, and the incidence of acute gastrointestinal injury was as high as 86.7% (130), and it often indicated a higher mortality rate (130–134). This suggested a vicious cycle between the intestinal damage caused by SARS-CoV-2 and the inflammatory response caused by it. In addition, almost half of the new coronavirus patients with gastrointestinal symptoms in a study developed after treatment, and were at increased risk of severe disease (135). Inappropriate treatment may also be a risk factor for inducing gastrointestinal symptoms. However, the research of Nobel, Yael R. showed that the appearance of gastrointestinal symptoms often indicated that the disease is “slow but not serious”, and gastrointestinal diseases will not hinder the host’s immune response to SARS-COV-2. The occurrence of gastrointestinal symptoms may be related to the faster reduction of viral load through the fecal route (136). Even among the many possible risk factors, the lack of gastrointestinal symptoms predicted reduced survival rates for all age groups (137). Patients with gastrointestinal symptoms when the test was positive had a significantly lower case fatality rate compared with those who were asymptomatic, but the course of the disease was longer (136, 138). Patients with only gastrointestinal symptoms had a milder illness and lower mortality than those with respiratory symptoms (139). This may be due to the low level of inflammation in the lamina propria cells of the gastrointestinal tract, the downregulation of inflammatory genes, and the reduction of major inflammatory proteins in the gastrointestinal tract, which weakened the inflammatory response caused by SARS-CoV-2 (140). Diarrhea was also an independent factor predicting lower mortality (141). On the other hand, some studies pointed out that the current results were not enough to prove that there was a significant correlation between the gastrointestinal symptoms of patients with the new coronavirus and the severity of the disease (142). The gastrointestinal symptoms of COVID-19 were common and had nothing to do with poor prognosis, the need for mechanical ventilation, and mortality (143, 144). To determine the relationship between gastrointestinal symptoms and disease prognosis, it was also necessary to exclude therapeutic effects, the indirect effects of cytokines, and the effects of drugs, as well as higher-level clinical research evidence (145). In this regard, the case–control study of Liu pointed out that compared with gastrointestinal symptoms, stool examination had a stronger correlation with patients’ severe and critical illness, so it provided more valuable information (146). Viral load also played an important role in assessing the severity of SARS-CoV-2 and other viral infections (51). Based on this, the most important thing to pay attention to is the early detection of potential infections, especially the isolated gastrointestinal tract. Patients with symptoms should take timely and effective preventive measures to prevent the hidden spread of the virus in the population.

## Summary

COVID-19 is the third coronavirus disease in this century. It has erupted globally since the end of 2019. The main symptoms of infection were fever, cough, and other lung symptoms. Some patients had gastrointestinal symptoms, such as diarrhea, abdominal pain, and vomiting. The main route of transmission is direct contact transmission, including droplet transmission and close contact transmission. Some patients had infectious SARS-CoV-2 in their feces, which suggested that SARS-CoV-2 could be transmitted through fecal–oral route. Gastrointestinal symptoms caused by COVID-19 occurred in patients with mild or severe infection, and their manifestations were diverse. Diarrhea and anorexia were the most common symptoms. Some serious diseases such as intestinal perforation and volvulus were also reported from time to time. It is difficult to draw a conclusion about the relationship between gastrointestinal symptoms and prognosis. The consensus is that the most important thing to note is to identify patients with gastrointestinal infections as the first manifestation. The direct causes of intestinal symptoms caused by SARS-CoV-2 were virus infection, cytokine release syndrome, or cytokine storm. Immune response disorder, intestinal flora disorder, vascular underwear cell damage, and thromboembolism were also the causes of severe gastrointestinal complications. Due to the existence of the gut–lung axis, the ecology of intestinal microbes is also of great significance to the development of pulmonary immunity and lung diseases. Therefore, we should also pay attention to the balance of the intestinal microorganisms, which will have a certain effect on the rehabilitation of patients and the reduction of normal human infections.

## Author Contributions

HZ, BS, QD, ZC, QZ and HL are responsible for writing the manuscript, and HZ is responsible for the revision of the manuscript. HZ and BS contributed equally to this work. ZS and WY are responsible for the editing of the manuscript. All authors contributed to the article and approved the submitted version.

## Funding

This study was supported by the National Natural Science Foundation of China (81972663 and 81560385), the Key Scientific Research Projects of Institutions of Higher Education in Henan Province (19A310024), the Medical Scientific and Technological Research Project of Henan Province (201702027), the Youth Innovation Fund Project of The First Affiliated Hospital of Zhengzhou University (YNQN2017035), the China Postdoctoral Science Foundation (2019T120648, 2017M610462), the National Natural Science Foundation of Henan Province (182300410342), and the Health Commission Technology Talents Overseas Training Project of Henan Province (2018140).

## Conflict of Interest

The authors declare that the research was conducted in the absence of any commercial or financial relationships that could be construed as a potential conflict of interest.

## Publisher’s Note

All claims expressed in this article are solely those of the authors and do not necessarily represent those of their affiliated organizations, or those of the publisher, the editors and the reviewers. Any product that may be evaluated in this article, or claim that may be made by its manufacturer, is not guaranteed or endorsed by the publisher.
